# Determination of Temporal Order among the Components of an Oscillatory System

**DOI:** 10.1371/journal.pone.0124842

**Published:** 2015-07-07

**Authors:** Sandra Barragán, Cristina Rueda, Miguel A. Fernández, Shyamal D. Peddada

**Affiliations:** 1 Departamento de Estadística e Investigación Operativa, Universidad de Valladolid, Valladolid, Spain; 2 Biostatistics Branch, NIEHS (NIH), Research Triangle Park, North Carolina, United States of America; University of Cambridge, UNITED KINGDOM

## Abstract

Oscillatory systems in biology are tightly regulated process where the individual components (e.g. genes) express in an orderly manner by virtue of their functions. The temporal order among the components of an oscillatory system may potentially be disrupted for various reasons (e.g. environmental factors). As a result some components of the system may go out of order or even cease to participate in the oscillatory process. In this article, we develop a novel framework to evaluate whether the temporal order is unchanged in different populations (or experimental conditions). We also develop methodology to estimate the order among the components with a suitable notion of “confidence.” Using publicly available data on *S. pombe*, *S. cerevisiae* and *Homo sapiens* we discover that the temporal order among the genes *cdc18*; *mik1*; *hhf1*; *hta2*; *fkh2* and *klp5* is evolutionarily conserved from yeast to humans.

## Introduction

Oscillatory systems arise naturally in biological sciences such as in, circadian biology [1–3], cell biology [4–9], endocrinology [10], metabolic cycle [11], evolutionary psychology [12, 13], motor behavior [14], and so on. An unperturbed oscillatory system is a tightly regulated temporal process with several components that execute their functions in an orderly manner like an orchestra. Thus a temporal order among the components is intrinsic to an oscillatory system. For example, it is well-known that our daily sleep and wake patterns lead to a sequence of biochemical events in the body in an orderly manner, such as breakdown of molecules to generate energy (catabolism) during the wake period and anabolism that takes place during the sleep period where growth of tissues occurs. Discussing the oscillations of individual neurons of the suprachiasmatic nuclei (SCN) in a 24 hour period, [15] describe the temporal order of circadian genes such as *Bmal1, Clock, Period, Cryptochrome, Rev-erb* [3]. The effect of sleep patterns on the temporal order of several circadian genes and consequently the effect on oxidative stress and metabolism was discussed in [16].

The common underlying question of scientific interest is to determine (relative) time to peak expression of genes participating in the oscillatory system [7, 12], i.e. to determine the underlying temporal order. A related question of interest is to understand the differences in the oscillatory systems of different populations or experimental groups such as; environmental conditions, species, organs within a species [17, 18], etc. Often raw expressions from time course experiments are used to make such inferences. For example studying circadian genes in various tissues in a whole animal and those in a cell line, [2] note that “relative phasing of core clock genes was estimated by visual inspection and plotted on a circular phase map.” Although such visual methods are easy to understand and implement, and widely used, they ignore uncertainty associated with the estimated values of angular parameters. Consequently it is not entirely surprising that there are disagreements in the literature regarding phases and phase order of various cell-cycle genes, even within the same species let alone across species [19].

Notice that, in this paper, we are not trying to establish which genes are periodic [20, 21] or to cluster genes according to their expression pattern [22, 23] but to see if the different phase angles assigned in different experiments to orthologs coming from several species are compatible with a common ordering of the phase angles of these genes across the species considered.

It is important to note that phase or time to peak expression of an oscillatory gene is a parameter on a unit circle and not on the real line. Consequently standard methods of analysis, such as the t-test or ANOVA, designed for real line data, cannot be used. Toy example in S1 File amplifies the problem of using such methods for angular data. Yet, they are commonly used in the literature [16], which may potentially result in incorrect or meaningless interpretations of the data.

**Fig 1:**
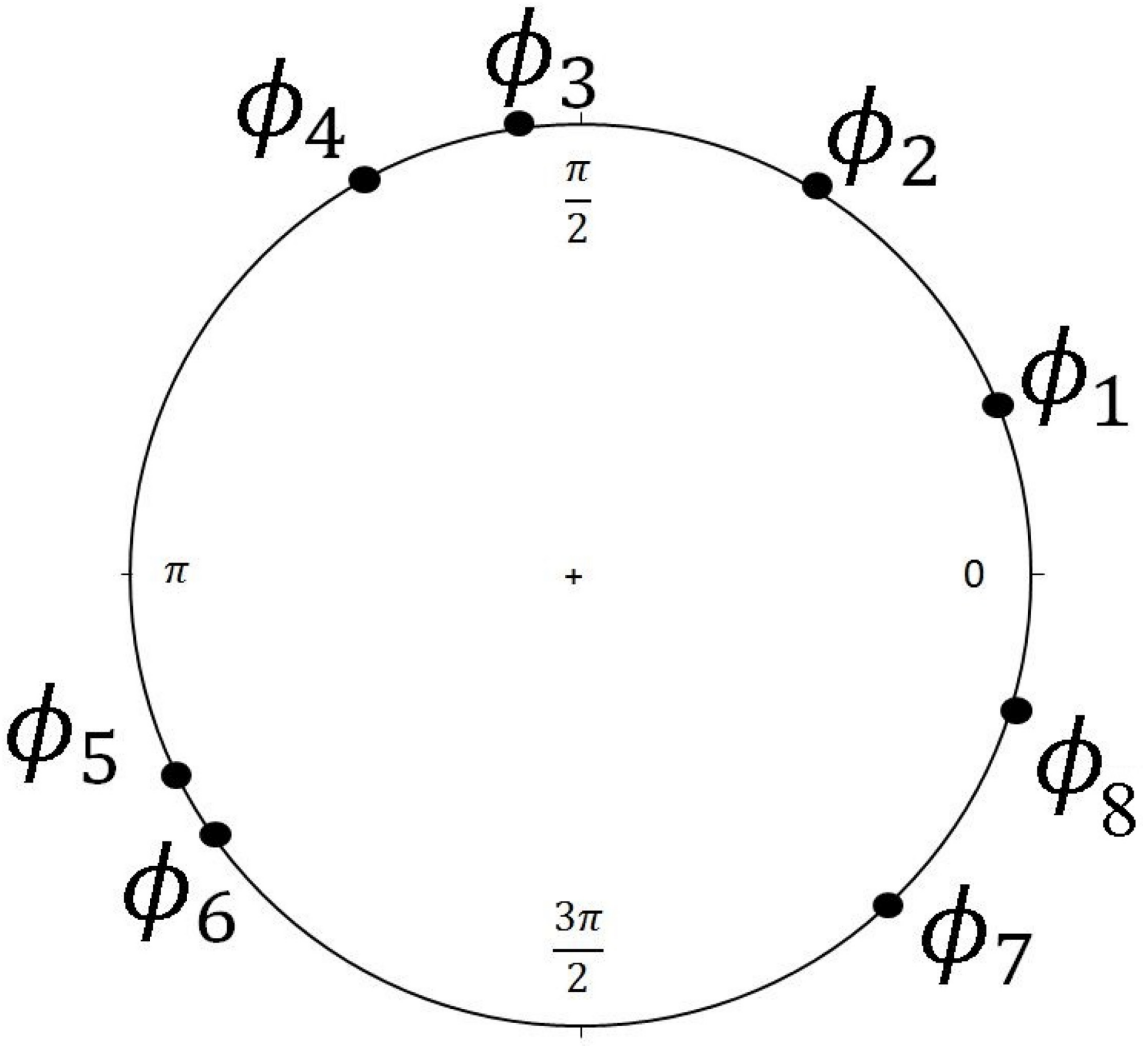
An illustration of the temporal order among genes *g*_1_, *g*_2_, …, *g*_8_ whose phase angles are in order along a circle (in counterclock-wise direction).

Analysis of angular data has a long history with well-developed theory and methodology documented in several books [24, 25]. Until recently much of the literature was developed for drawing inferences on individual parameters, but not for studying order among a set of angular parameters (e.g. phases of a system of oscillatory genes), which is the focus of this article. More precisely, suppose an oscillatory system consists of genes, *g*_1_, *g*_2_, *g*_3_, …, *g*_8_, with phase angles *ϕ*_1_, *ϕ*_2_, …, *ϕ*_8_, respectively. Then a researcher is typically interested in determining the circular order (temporal order) among these phase angles. For example, determine whether *g*_1_ peaks before *g*_2_ which peaks before *g*_3_, etc. *g*_7_ peaks before *g*_8_ and *g*_8_ before *g*_1_. Mathematically, determine if *ϕ*_1_ precedes *ϕ*_2_ which precedes *ϕ*_3_ and so on *ϕ*_7_ precedes *ϕ*_8_ which in turn precedes *ϕ*_1_ around the unit circle (e.g. Fig 1). We shall denote the order by *ϕ*_1_ ≼ *ϕ*_2_ ≼ ⋯ ≼ *ϕ*_7_ ≼ *ϕ*_8_ ≼ *ϕ*_1_.

For two or more study groups (e.g. organs or species, etc.), researchers are typically interested in testing whether the temporal order of a set of oscillatory genes is conserved. If so, they are interested in discovering the common temporal order with an estimate of confidence. In this article we introduce a statistical framework to address such problems. We illustrate the methodology by discovering a temporal order among a core set of cell cycle genes that is conserved from yeast to humans. Although the methodology described in this paper is suitable for any oscillatory system, for convenience of exposition we use cell-cycle terminology.

The temporal order derived by the proposed methodology could potentially help biologists to discover or explore novel regulatory relationships among the genes in the oscillatory system. Thus our methodology can potentially lead to new hypotheses for biologists to study.

## Materials and Methods

### Estimation of temporal order

Before describing the methodology to test hypothesis regarding the circular order among a set of oscillatory genes, we discuss the problem of estimating their common unknown circular order (assuming it exists). Using this estimator we then develop a statistical procedure to test the null hypothesis that a given set of oscillatory genes in two or more study groups (or populations) share the same temporal order.

In addition to estimating the unknown phase angles *ϕ*_1_, *ϕ*_2_, …, *ϕ*_*n*_ the goal is also to estimate the true relative order among them, denoted by *O* = (*o*_1_, *o*_2_, …, *o*_*n*_), where *ϕ*_*o*_1__ ≼ *ϕ*_*o*_2__ ≼ ⋯ ≼ *ϕ*_*o*_*n*__ ≼ *ϕ*_*o*_1__. Note that *O* is rotation invariant. Thus by moving the pole around the circle between each consecutive pair of angular parameters, we obtain *n* possible equivalent orders to *O*. The goal is to estimate *O* using data obtained from *p* experiments. We will denote the estimator of *O* as $\tilde{O}$ and is obtained by the procedure explained below.

Typically, researchers conduct time course gene expression studies to obtain the phases of each cell-cycle gene. For the *i* ^*th*^ gene in the *j* ^*th*^ experiment, let *θ*_*ij*_ denote the estimate of phase angle *ϕ*_*i*_ obtained by using the Random Periods Model, RPM [26]. Since the estimates obtained from RPM are not constrained by any order among the phase angles, they are called the unconstrained estimators. Accordingly, let

$$\Theta_{j}={\left(\theta_{1j},\ldots ,\theta_{ij},\ldots ,\theta_{nj}\right)}^{\prime }\;\forall j=1,\cdots ,p,$$

denote the vector of RPM estimators of (*ϕ*_1_, *ϕ*_2_, …, *ϕ*_*n*_)′ obtained from the *j* ^*th*^ experiment. Stacking all such estimators for the *p* experiments together, we have Θ = [Θ_1_, …, Θ_*p*_].

We estimate *O* using the minimum distance principle as follows. Let O denote the set of all possible orders among *ϕ*_1_, *ϕ*_2_, …, *ϕ*_*n*_. Using the data from the *j* ^*th*^ experiment, under a given order O ∈ 𝔒, let ${\tilde{\Theta }}_{j}^{\left(\text{O}\right)}={\left({\tilde{\theta }}_{1j}^{\left(\text{O}\right)},{\tilde{\theta }}_{2j}^{\left(\text{O}\right)},\ldots ,{\tilde{\theta }}_{nj}^{\left(\text{O}\right)}\right)}^{\prime }$ denote the circular isotonic regression estimator (CIRE) of *ϕ*_1_, *ϕ*_2_, …, *ϕ*_*n*_ under the circular order constraint O [8].

As in [4] and [8] the sum of circular errors (SCE), which serves as the distance between Θ_*j*_ and the order *O*, is defined as follows.

**Definition 1** *The Sum of Circular Errors (SCE) corresponding to circular order* O *for data in the j^th^ experiment*, Θ_*j*_ = (*θ*_1*j*_, *θ*_2*j*_, …, *θ_nj_*)′, *is given by:*

$$SCE\left(\Theta_{j},{\tilde{\Theta }}_{j}^{\left(\text{O}\right)}\right)=\sum_{i=1}^{n}\left\{1-\cos\left(\theta_{ij}-{\tilde{\theta }}_{ij}^{\left(\text{O}\right)}\right)\right\}.$$

For a given order O, its mean sum of circular errors (MSCE) over all *p* experiments is given by:

$$d\left(\Theta ,\text{O}\right)=MSCE\left(\Theta ,{\tilde{\Theta }}^{\left(\text{O}\right)}\right)=\sum_{j=1}^{p}\omega_{j}\frac{1}{n}SCE\left(\Theta_{j},{\tilde{\Theta }}_{j}^{\left(\text{O}\right)}\right),$$ (1)

where *ω*_*j*_ is the weight associated with *j* ^*th*^ experiment. Suppose *θ*_*ij*_ ∼ *M*(*ϕ*_*i*_, *κ*_*j*_) where *M* denotes the von-Mises distribution with angular mean *ϕ*_*i*_ and concentration parameter *κ*_*j*_ (known), then we define $\omega_{j}=\frac{\kappa_{j}}{\sum_{j=1}^{p}\kappa_{j}}$.

The optimum circular order can be obtained by solving the following minimization problem:

$$\min_{\text{O}\in 𝔒}\;d\left(\Theta ,\text{O}\right)=\min_{\text{O}\in 𝔒}\sum_{j=1}^{p}\omega_{j}\frac{1}{n}SCE\left(\Theta_{j},{\tilde{\Theta }}_{j}^{\left(\text{O}\right)}\right).$$ (2)

The above problem resembles the classical problem of determining the “true” order or ranks among *n* objects using the scores assigned by *p* independent “judges”. For example, suppose there are *n* gymnasts competing in an event and there are *p* judges assigning scores to each of the contestants. The goal is to estimate the true rank among the *n* contestants using the scores assigned by the *p* judges. Although this NP-hard problem [27] is well-studied in the Euclidean space [28–31], it has not been discussed for other geometries such as the circle. Due to the underlying geometry, the Euclidean space based methods cannot be directly applied here.

Since the above formulation is NP hard even for real line data, we obtain an approximate solution by reformulating Eq (2) as a traveling salesman problem (TSP) which is known to be NP-complete [32, 33].

The TSP is well-studied in the graph theory literature [34–36] and is formulated using a weighted graph which is a triple consisting a set of nodes, a set of edges and a cost associated with each edge. The purpose of TSP is to determine the tour with minimum total cost, where a tour is the path traveled by a salesman such that all nodes are visited and each node is visited exactly once. In our application genes are the nodes, edge is the path between two genes and a tour is a circular order among the genes. For the simulations we have performed with a moderate number of elements to be ordered (notice that, as usual in these problems, the optimum value cannot be computed in a reasonable time when the number of elements increases), this TSP approach performed very well so that we expect the tour with minimum total cost to be a good approximation to our original problem Eq (2).

To determine the tour with minimum total cost we first define the total cost of traveling between nodes *h* and *k* in the *p* experiments (*E*_*hk*_) as the weighted sum $E_{hk}=\sum_{j=1}^{p}\omega_{j}E_{hk}^{j}$, where $E_{hk}^{j}$ is the cost in the *j* ^*th*^ experiment. For each *j*, the cost $E_{hk}^{j}$ is defined through a measure of distance between the nodes *h* and *k*. A common measure of distance between a pair of points on a unit circle is 1 − cos(*θ*_*kj*_ − *θ*_*hj*_) [25]. This measure is symmetric but cell-cycle is a biological process where the functional relations between genes are not symmetric. Without loss of generality the sequential order of events (or phases) of cell-cycle may be represented in the counter-clockwise direction around the unit circle. For this reason we define distances asymmetrically, depending upon whether the salesman is traveling counter-clockwise (*d*_1_) or clockwise (*d*_2_) as follows:

$$d_{1}^{j}\left(h,k\right)=\{\begin{array}{cc} 1-\cos(\theta_{kj}-\theta_{hj})\; & \text{if}\;0\leq \theta_{kj}-\theta_{hj}\leq \pi \\ 3-\cos(\theta_{kj}-\theta_{hj}-\pi )\; & \text{if}\;\pi <\theta_{kj}-\theta_{hj}\leq 2\pi , \end{array}$$

$$d_{2}^{j}\left(h,k\right)=\{\begin{array}{cc} 3-\cos(\theta_{kj}-\theta_{hj}-\pi )\; & \text{if}\;0\leq \theta_{kj}-\theta_{hj}\leq \pi \\ 1-\cos(\theta_{kj}-\theta_{hj})\; & \text{if}\;\pi <\theta_{kj}-\theta_{hj}\leq 2\pi . \end{array}$$

Asymmetric distances are common in the application of TSP and are widely studied [37]. Using the above distances, we define the cost of traveling between the nodes *h* and *k* in the experiment *j* as follows:

$$E_{hk}^{j}=\min\left(d_{1}^{j},\alpha d_{2}^{j}\right),$$

where *α* represents the penalty for traveling in the clockwise direction. Based on extensive simulation studies using different values of *α*, we found *α* = 3 provided the best results and hence we use this value throughout the paper.

Let *X* denote an *n* × *n* matrix where *x*_*hk*_ = 1 if the salesman travels directly from node *h* to node *k*, otherwise let *x*_*hk*_ = 0. No sub-tours are allowed. Let 𝓧 denote the collection of all such matrices which represent a tour. Then, TSP reduces to solving the following minimization problem

$$\min_{X\in 𝓧}\sum_{hk}\;x_{hk}E_{hk}=\min_{X\in 𝓧}\sum_{j=1}^{p}\omega_{j}\;(\sum_{hk}\;x_{hk}E_{hk}^{j}).$$ (3)

We denote as ${\tilde{X}}^{0}$ the solution of Eq (3). The resulting order among the nodes denoted as ${\tilde{O}}^{0}$ is taken to be an approximate solution to Eq (2). To improve this approximation, we refine it by eliminating any local bumps (i.e misalignment of order). The chances of misalignment of order can occur locally as the number of nodes (genes) increases or as some nodes get closer to each other. We accomplish this by modifying the *Local Kemenization* algorithm that was originally developed by [38] for the Euclidean data to the present context of circular data. We call the resulting algorithm the *Circular Local Minimization* algorithm. It consists of checking each consecutive triple (*h*, *k*, *l*) of adjacent elements in ${\tilde{O}}^{0}$ (while preserving the estimated circular order among rest of the elements) to see if a permutation of *o*_*h*_, *o*_*k*_, *o*_*l*_ improves the result. Namely, we calculate the MSCE as defined in Eq (1) between the possible new circular order, with the permutation, and the data. If the new MSCE is smaller then the circular order is appropriately changed. The resulting refined estimate is $\tilde{O}$.

### Comparison of temporal orders

Suppose there are *S* experimental groups and *n* genes in each group that oscillate. Let *O*_*s*_, *s* = 1, 2, …, *S*, denote the order among the phase angles of the *n* genes in the *s* ^*th*^ group. Then the problem of interest is to test:

$$\begin{array}{c} H_{0}\;:\;O_{1}=O_{2}=\ldots =O_{S}=O_{*}\\ H_{1}\;:\;H_{0}\;\text{not}\;\text{true}. \end{array}$$

The equality sign “=” in the null hypothesis denotes “identical circular order” which would be represented by O_*_. Corresponding to the *s* ^*th*^ group, *s* = 1, 2, …, *S*, suppose there are *p*_*s*_ experiments. Let $P=\sum_{s=1}^{S}p_{s}$ denote the total number of experiments. Then the above hypothesis can be tested along the lines of classical analysis of variance (ANOVA). Let ${\tilde{O}}_{s}$ denote the estimated order obtained with the experiments from the *s* ^*th*^ group and ${\tilde{O}}_{*}$ denotes the estimated common order under the above null hypothesis obtained by using the data from *P* experiments combining data from all *S* groups.

Let $d(\Theta^{\left(s\right)},{\tilde{O}}_{s})=\sum_{j=1}^{p_{s}}\;\omega_{j}^{s}\frac{1}{n}SCE(\Theta_{j}^{\left(s\right)},{\tilde{\Theta }}_{j}^{\left({\tilde{O}}_{s}\right)})$ denote the corresponding value of the objective function Eq (2) for the experiments in the *s* ^*th*^ group. Here $\omega_{j}^{s}$ denotes the weight corresponding to the *j* ^*th*^ experiment in the *s* ^*th*^ experimental group. Adding over all *S* experimental groups we have the following which resembles the *within groups variability*, $\sum_{s=1}^{S}d(\Theta^{\left(s\right)},{\tilde{O}}_{s})$.

Let $d(\Theta^{\left(.\right)},{\tilde{O}}_{*})=\sum_{s=1}^{S}\sum_{j=1}^{p_{s}}\omega_{j}^{s}\frac{1}{n}SCE(\Theta_{j}^{\left(s\right)},{\tilde{\Theta }}_{j}^{\left({\tilde{O}}_{*}\right)})$ denote the corresponding value of the objective function Eq (2) using the data for all *P* experiments. This expression resembles the *global variability*. Hence, resembling the classical ANOVA, one may consider any monotonic function of the following test statistic for testing above null hypothesis:

$$T=\frac{d\left(\Theta^{\left(.\right)},{\tilde{O}}_{*}\right)-\sum_{s=1}^{S}d\left(\Theta^{\left(s\right)},{\tilde{O}}_{s}\right)}{d(\Theta^{\left(.\right)},{\tilde{O}}_{*})}.$$

Since not all species (in this case the experimental groups) are represented by equal number of experiments and not all experiments are subject to same experimental error/noise, we use a “weighted” resampling method to derive the p-values based on *T* that takes into account all such features of the data. The goal is to create artificial species that resemble the original species in terms of the expected proportions of experiments within each species. We therefore select experiments randomly with replacement and equal probabilities per species and per experiment within species. Thus each experiment in the *s* ^*th*^ species has a probability 1/(*Sp*_*s*_) of selection. Under this sampling scheme we select *P* random experiments with replacement from the *P* actual experiments and assign the first *p*_1_ to artificial species 1, the next *p*_2_ to artificial species 2 etc. The weights per experiment are suitably calculated with each resample. Extensive simulation experiments, under a variety of configurations of phase angles and the order among phase angles were conducted to evaluate the Type I error rate of the proposed resampling scheme. Based on our results, detailed in the S3 File, we discover that the proposed resampling procedure yields honest statistical test in the sense that the estimated Type I error never exceeds the nominal rate of 0.05 by more than a standard error. Furthermore, the proposed methodology enjoys very high power even under minor departures from the null hypothesis.

For genes identified to satisfy a common global order, we use the above resampling procedure in combination with the estimation procedure described in the previous section to estimate the common global partial order with confidence as follows. We take the union of most frequent orders coherent with the common global order to deduce the global partial order. The sum of the frequencies of those orders relative to the total number of resamples provides the confidence coefficient. To illustrate the methodology, suppose *g*_1_, *g*_2_, …, *g*_5_ are determined to satisfy common global order among 3 species according to the above test. Suppose we obtain 1000 samples according to the above resampling scheme and for 600 of them the global order is *g*_1_ ≼ *g*_3_ ≼ *g*_4_ ≼ *g*_5_ ≼ *g*_2_ ≼ *g*_1_ and for 300 of them the global order is *g*_1_ ≼ *g*_3_ ≼ *g*_4_ ≼ *g*_2_ ≼ *g*_5_ ≼ *g*_1_. For the remaining 100 resamples, suppose the global orders are arbitrarily distributed among the other possible orders. Note that in a large proportion of resampled data the order between *g*_2_ and *g*_5_ is not consistent. In 60% of the resamples *g*_5_ precedes *g*_2_ whereas in 30% of the resamples the order is reversed. In such cases we assign a “partial order” to indicate that the order between *g*_2_ and *g*_5_ is undetermined. Thus the global partial order in this toy example is given by *g*_1_ ≼ *g*_3_ ≼ *g*_4_ ≼ {*g*_5_, *g*_2_} ≼ *g*_1_ with 90% confidence.

## Results

### Motivation and background

Since cell division cycle is an essential process for growth and development of all living organisms, there has been considerable interest among cell biologists to identify cell-cycle genes that are evolutionarily conserved in their functions across multiple species [5–7, 9, 19, 39]. Cell-cycle is a well-coordinated process where events must take place in an orderly fashion for a successful cell division. Hence genes participating in the cell division cycle express in an order according to their function. Throughout this section we focus on only those cell-cycle genes that have a periodic or oscillatory expression (i.e. dynamic) and not those genes that participate in cell division cycle but are static in their expression. Thus a question of interest is to determine, among periodically expressed genes, whether the order of peak expression is evolutionarily conserved. Such questions were extensively discussed and debated during the past decade using gene expression data obtained from budding yeast (*S. cerevisiae*), fission yeast (*S. pombe*) and human Hela cell [5–7, 9]. There are several biological complexities associated with such questions. Firstly, there is considerable disagreement in the literature on the number of genes that are periodic in multiple species [5–7, 9]. As noted in [19], there is considerable disagreement among studies even within the same species. They observed that the three recent studies on the fission yeast [6, 7, 9], together identified about 1400 genes to be periodic, yet only about 10% of these genes were common to all three studies and only about 30% were common to any pair of studies. Given that there is such a large disagreement among studies even within the same species, it is not surprising that there are diverse opinions regarding the number of genes that are periodic in the two species of yeast, namely, the budding yeast (*S. cerevisiae*) and the fission yeast (*S.pombe*). Conservative estimates of the number of genes that are periodic in both species of yeast is about 35 and the number that are periodic in the two yeasts and humans is about 11, see [4]. Furthermore, among genes that were identified to be periodic within the same species by different studies, there are disagreements regarding the phase of peak expression of some genes. For example, [40] assigned *E2F5*, an important transcription factor, to G2/M phase whereas according [41, 42] it peaks during G1/S phase. In the case of fission yeast, [7] assigned *cdc18*, a gene whose protein is essential for the initiation of DNA replication, to G1/S phase whereas [6] as well as cyclebase (www.cyclebase.org) [43] assigned the gene to peak in the M phase. It has been a challenging problem to determine if the phase of a cell-cycle gene is conserved evolutionarily. This is partly because, in addition to the above mentioned issues, the amount of time a cell spends in a given phase is not evolutionarily conserved. For example, a fission yeast cell spends more than 70% of its time in the G2 phase while a budding yeast cell spends about equal time in all phases.

Secondly, a gene needs to be converted into protein before it performs its function. So, even if a cell-cycle gene’s function is conserved evolutionarily, its phase may not necessarily be. Thirdly, for a given gene in a particular species it may have multiple orthologs in other species, hence it is a many to many mapping and not a one to one mapping. Since not all orthologs are equally periodic (using the periodicity measure provided in cyclebase), it is a challenging problem to discuss conservation of phase across all orthologs of a gene. Thus it is not surprising for [5] to state that these analysis reveal that periodic expression is poorly conserved at the level of individual genes: conserved periodic expression across the organisms considered is observed in only five cases and for only two of these is the timing conserved as well, namely histones *H*2*A* and *H*4.

Although, for the above reasons, it may be difficult to ascertain if the phase of a cell-cycle gene is evolutionarily conserved, it may be plausible that the relative order among a collection of cell cycle genes may be evolutionarily conserved. An attempt was made in [4] to answer this question by testing the null hypothesis that the relative order of a subset of cell-cycle genes is conserved between fission yeast and budding yeast. They also performed a similar test between fission yeast and human Hela cells. A drawback with their methodology is that they assume the relative order of cell-cycle genes is known with certainty in one of the two species that are being compared. This is analogous to the “one sample test”. Furthermore their methodology is not suitable for testing for the order in more than two species. The present methodology, however, overcomes those deficiencies. In this section we illustrate the methodology by analyzing the phase angle data on 11 cell-cycle genes that are known to be periodic in the 3 organisms. In addition to testing whether the relative order is conserved among the 3 species, we discover the order along with an estimate of confidence in the estimated order. Before proceeding further, we like to remark that [4] do not draw distinctions between orthologs and paralogs since their goal was to determine conservation of order among periodic genes. Again, as noted earlier, not all orthologs of a gene across species are equally periodic -some may not be periodic at all. In such cases, rather asking the question if the relative order of a gene is conserved across all species for all orthologs of a gene, we limit only to the most periodic ortholog (as determined by databases pombase and cyclebase). As in [4] we use the periodicity rank provided in cyclebase. The only exception is human ortholog of *ace*2, which we took to be ZNF367.

Remark: For illustration purposes, in this section we are only considering the case where one is interested in testing the order *g*_1_ ≼ *g*_2_ ≼ … ≼ *g*_*n*_ ≼ *g*_1_ among a set of singleton genes *g*_1_, *g*_2_, …, *g*_*n*_. However, as seen from the results of the analysis provided in the next section, for a given data set, it is possible that our algorithm may declare a subset of these genes to have same order relative to other genes (see Eq (5) in the next section).

If one is interested in the testing for the conservation order of groups of genes (or orthologs) rather than singletons as above, then our methodology can be easily extended to test orders among groups of genes. More precisely, our methodology can be extended to test the order

$$\left\{g_{11},g_{12},\ldots ,g_{1r_{1}}\right\}≼\left\{g_{21},g_{22},\ldots ,g_{2r_{2}}\right\}≼\ldots ≼\left\{g_{n1},g_{n2},\ldots ,g_{nr_{n}}\right\},$$

where the order among the genes (or orthologs) within {} is irrelevant but as a group they are ordered with the previous and the next group. Thus our method can handle situations where a biologist may be interested in studying the relative order of groups of cell-cycle genes. For example, several cell-cycle genes encode proteins that make up large protein assemblies and since all of the subunits within each assembly would be needed for the function of that assembly to be carried out, one may be interested in testing for the order among such large assemblies and not interested in the order among the elements within each assembly.

### Determination of the common temporal order across species

We used the publicly available time course gene expression microarray data on humans (Hela cell), the budding yeast and fission yeast. Specifically, we used the four human data obtained from [40]; six budding yeast data (one from [44], another from [45], two from [46] and two from [20] and ten fission yeast data (five by [9], three by [6] and two by [7]. Thus we had access to data from 20 experiments conducted on 3 different species. We focused on the expression of 11 cell-cycle genes that are periodic in all 3 species (see Table 1). We estimated the phase angle of each gene within each experiment by fitting the RPM [26]. These estimates, known as the unconstrained estimates because they are obtained with no constraints of the phase angles, are reported in Table A in S2 File. The *κ*_*j*_ values used to determine the *ω*_*j*_ weights have been obtained using the procedure developed in [4] and appear in Table B in S2 File.

**Table 1:** Evolutionarily conserved human cell-cycle genes along with corresponding *S. pombe* and *S. cerevisiae* orthologs.

Genes	*S. pombe*	*S. cerevisiae*	*Humans*
1	*ace2*	*ACE2*	*ZNF367*
2	*cdc18*	*cdc18*	*CDC6*
3	*mik1*	*SWE1*	*PKMYT1*
4	*hhf1*	*HHF1*	*HIST2H4B*
5	*hta2*	*HTA2*	*H2AFX*
6	*fkh2*	*FKH1*	*FOXM1*
7	*klp5*	*KIP3*	*KIF10*
8	*cig2*	*CLB1*	*CCNB1*
9	*plo1*	*CDC5*	*PLK1*
10	*slp1*	*CDC20*	*CDC20*
11	*rad21*	*MCD1*	*RAD21*

To determine whether the temporal order is conserved across the 3 species, we first tested the following hypotheses using all 11 genes:

$$\begin{array}{c} H_{0}\;:\;O_{fission\;yeast}=O_{budding\;yeast}=O_{humans}\\ H_{1}\;:\;H_{0}\;\text{not}\;\text{true}. \end{array}$$ (4)

Our resampling procedure rejected the null hypothesis with a p-value of 0.0045. This suggests that at least one of the 11 genes was out of order in at least one pair of species. In order to determine a maximum size subset of genes for which the three species share a common order we applied the forward procedure described in the S4 File.

The process ended with the 6 genes, *klp*5, *fkh*2, *cdc*18, *mik*1, *hhf*1 and *hta*2, that failed to reject the null hypothesis with a p-value of 0.488 (see Table 2). Thus we conclude that the temporal order among these genes is evolutionary conserved from yeast to humans with the following partial order,

$$\begin{array}{c} cdc18≼mik1≼hhf1≼hta2≼\{fkh2,klp5\}≼cdc18 \end{array}$$ (5)

**Table 2:** Summary of the 4 comparisons.

	#genes	P-value	Confidence Coefficient
All 3 species together	6	0.488	100%
*S. pombe—S. cerevisiae*	10	0.336	72.31%
*S. pombe—Humans*	8	0.436	92.6%
*S. cerevisiae—Humans*	6	0.119	99.15%

Using the estimation and the resampling methodology described in this article, we estimated that the confidence of this partial order Eq (5) is 100%. The most frequent simple circular order *cdc*18 ≼ *mik*1 ≼ *hhf*1 ≼ *hta*2 ≼ *klp*5 ≼ *fkh*2 ≼ *cdc*18 had an estimated confidence coefficient of 76.06%.

The two yeasts shared a common ancestor nearly a billion years ago and neither is closer to human beings more than the other [47]. However, according to [48] and [49], while *S. pombe* and metazoan cell-cycle genes retained some of the functions from their common ancestor, the budding yeast cell-cycle genes may have lost them. In fact, relative to *S. cerevisiae* there are proportionally more *S. pombe* genes conserved in metazoans [48, 50]. There are other similarities between *S. pombe* and higher order animals including stress response pathways. For a review one may refer to [47–50]. In view of the above discussion, we performed pairwise comparisons between the 3 species starting with the 6 genes discovered above.

The pairwise forward selection analysis between the two yeasts (*S. pombe* and *S. cerevisiae*) revealed that the relative order of peak expression among 10 out of the 11 genes was conserved with an associated p-value of 0.336. The relative was determined to be *cdc*18 ≼ *rad*21 ≼ *mik*1 ≼ {*ace*2, *hhf*1, *hta*2, *cig*2} ≼ {*fhk*2, *klp*5} ≼ *slp*1 ≼ *cdc*18 with a confidence coefficient of 72.31%. In the case of *S. pombe* and *humans* the relative order was conserved among 8 of the 11 genes with an associated p-value of 0.436, with relative order {*ace*2, *cdc*18} ≼ *mik*1 ≼ *hhf*1 ≼ *hta*2 ≼ *plo*1 ≼ {*fhk*2, *klp*5} ≼ {*ace*2, *cdc*18}. The confidence coefficient associated with this order was estimated to be 92.6%. However, in the case of *S. cerevisiae* and *humans* we discovered that the order conserved only among the original 6 genes whose order was conserved among the 3 species, namely, *cdc*18, *mik*1, *hhf*1, *hta*2, *klp*5 and *fkh*2. Thus, we did not find any additional genes unlike the other 2 pairwise analyses. The p-value associated with these 6 genes in the *S. cerevisiae* and *humans* pair was 0.119 and the relative order was essentially same as when all three species were considered together but slightly perturbed. The estimated relative order among these 6 genes in the pair *S. cerevisiae* and *humans* was estimated to be *cdc*18 ≼ *mik*1 ≼ *hhf*1 ≼ *hta*2 ≼ {*fkh*2, *klp*5} ≼ *cdc*18 with a confidence coefficient of 99.15%. These results are summarized in Table 2. Full details of each of the steps in the procedure can be found in the Supporting Information.

Using published phases of these 6 genes in the literature, we summarize the phases of these 6 genes in the 3 species in Table 3. Note that while the phase order of the 6 genes is same across the 3 species their phases are not same across species.

**Table 3:** Phases of the 6 cell-cycle genes whose circular ordered is conserved in the 3 species according to www.cyclebase.org.

*S. pombe*		*S. cerevisiae*		*Humans*	
Gene	Phase	Gene	Phase	Gene	Phase
*klp5*	G2	*KIP3*	S/G2	*KIF10*	S
*fkh2*	G2	*FKH1*	G2/M	*FOXM1*	S/G2
*cdc18*	G1/S	*cdc18*	M	*CDC6*	M
*mik1*	S	*SWE1*	M	*PKMYT1*	G1/S
*hhf1*	S	*HHF1*	G1/S	*HIST2H4B*	S
*hta2*	S/G2	*HTA2*	G1/S	*H2AFX*	S

In the case of the two yeasts it is well known that the yeast orthologs of *fkh*2 and *ace*2 participate in a regulatory network loop where *fkh*2 regulates the expression of *ace*2 which in turn regulates *fkh*2 [51]. Furthermore *fkh2*, the *S. pombe* ortholog of *fkh*2, is one of the regulators of the Cdc15 clusters which peak in late G2 or M phase. In fact, according to [6] its expression peaks prior to 94% of the genes in the Cdc15 cluster, implying that it potentially regulates most of the genes in the cluster. Gene *ace2*, belongs to the Eng1 cluster which contains genes that regulate cell separation. These genes peak after the Cdc15 cluster of genes.

Interaction between the proteins of *cdc*18 and *mik*1 are well-known [52]. Furthermore, according to the Human Protein-Protein Interaction Prediction software [53, 54], the proteins *cdc*18 and *mik*1 are highly interactive. The probability that they interact with each other is 17.80 times the probability that they do not. Thus our method not only validates some of the well-known relationships and interactions but also provides the direction of the interaction, suggesting that possibly one gene regulates the other which may lead to new hypotheses for biologists to investigate.

## Discussion

Often biological processes involve complex network of inter-relationships among the components of the process (e.g. genes). Biologists have been interested in deriving such networks and using them for drawing inferences regarding the underlying biological process. In the case of an oscillatory system, such as the cell-cycle or circadian clock, these networks are intrinsically dynamic in nature with the system going through different states or phases (e.g. phases in cell-cycle) over time before returning to the original state. At each state, due to the underlying biology, a subset of the components plays a prominent role. For example, only those genes that are involved in DNA synthesis are likely to express during the S-phase of the cell-cycle and the others may not. However, once S-phase is completed, the next wave of genes that are involved in the *G*2 phase express, and so on. It is of interest for biologist to understand the temporal order of how genes regulate each other as the cell goes through various phases. Thus, in an oscillatory system it is of interest to determine the temporal order among the components. Because of the structure of oscillatory system, underlying statistical parameters of interest (e.g. phase angles of cell-cycle genes) are points on a unit circle and not the entire Euclidean space. Focus of this research is to determine the temporal order with confidence and to compare the temporal orders among various study groups. Because of the underlying geometry of the circle, standard Euclidean space based methods are not suitable and until [4] there did not exist any rigorous statistical framework to analyze such data. Although [4] take important first step towards this problem, their methodology cannot be used to estimate the underlying order among the components. Secondly, their methodology does not allow a researcher to simultaneously test for the equality of the order among 3 or more populations. Lastly, when comparing two populations, their methodology assumes that the order of expression among the components of one of the populations is known with certainty, an unreasonable assumption in practice. We not only overcome the above deficiencies of [4] but we also provide a novel method to estimate the common temporal order among a set of oscillatory genes across multiple populations, along with the associated confidence coefficient. Using the proposed methodology we successfully demonstrated that the temporal order of 6 cell-cycle genes is conserved in the two species of yeast and the humans. The proposed methodology can potentially be extended to develop dynamic networks for oscillatory systems where a biologist may be interested in not only inferring gene networks at a given time point but draw inferences across time points.

## Supporting Information

S1 File Angular mean versus Arithmetic mean for circular data. (PDF)

S2 File Unconstrained estimates of phase angles and the concentration parameters. (PDF)

S3 File Operating characteristics of the test statistic. (PDF)

S4 File Gene forward selection procedure and results. (PDF)
