# Supplementary material for: Determination of Temporal Order among the Components of an Oscillatory System
Source: PLoS One. 2015 Jul 7;10(7):e0124842. doi: 10.1371/journal.pone.0124842 (PMC4495067; doi:10.1371/journal.pone.0124842)
Supplement: S1 File — (PDF) [file pone.0124842.s001.pdf]

Angular mean versus Arithmetic mean for circular data

The left (right) panel of Figure A corresponds to the toy data in the left (right) panel of Table A. From the left panels we see that although the angular means are identical, the arithmetic means are poles apart. Thus the t-test, that uses the arithmetic mean, is likely to falsely declare a significant difference between the control and treated groups. From the right panels we observe just the opposite. Although the control and treated groups are poles apart in their angular means, the arithmetic means do not differ (180° each). Thus in this case the t-test is likely to falsely find no significant difference between the control and treated groups although they are pole apart.

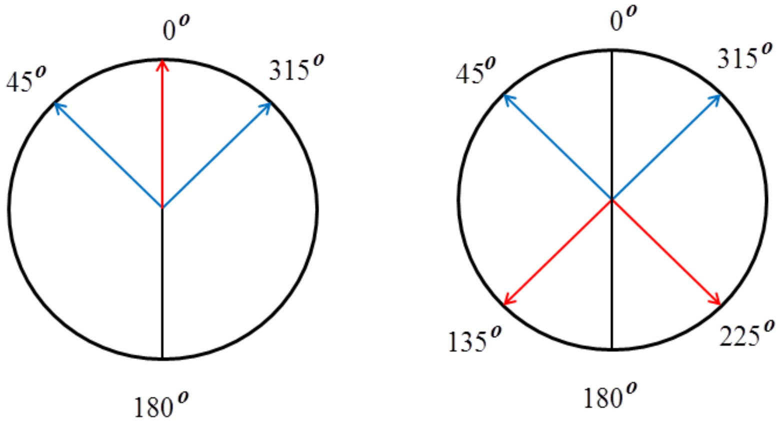

Figure A: Phase angles of samples from treatment group (red arrows) and the control group (blue arrows). Left panel corresponds to no difference in the angular mean directions while the right panel corresponds to the case where angular means are pointing in the opposite directions.

Table A: Observed phase angles for treatment and control samples along with arithmetic mean and angular mean directions.

Sample	Control	Treatment	Control	Treatment
Sample 1	45	0	45	135
Sample 2	315	0	315	225
Arithmetic mean	**180**	**0**	**180**	**180**
Angular mean	**0**	**0**	**0**	**180**
