# Supplementary material for: Determination of Temporal Order among the Components of an Oscillatory System
Source: PLoS One. 2015 Jul 7;10(7):e0124842. doi: 10.1371/journal.pone.0124842 (PMC4495067; doi:10.1371/journal.pone.0124842)
Supplement: S2 File — (PDF) [file pone.0124842.s002.pdf]

Unconstrained estimates of phase angles and the concentration parameters

Since the time course data are potentially subject to outliers and influential observation, we fitted the RPM model (Liu et al. (2004)) under the L_1 to obtain the unconstrained estimators of the phase angles of the 11 genes in the 20 experiments. We also estimated the values of the concentration parameter κ for the 20 experiments using the procedure developed in Fernández et al. (2012). Results are summarized in Tables A and B.

Table A: Unconstrained estimates of phase angles obtained using the Random Periods Model.

Species	Experiment	*ace2*	*cdc18*	*mik1*	*hhf1*	*hta2*	*fkh2*	*klp5*	*cig2*	*plo1*	*slp1*	*rad21*
S. pombe	1- Oliva et al., 2005 cdc	5.69	0.00	5.77	0.68	1.12	6.21	1.25	5.86	0.75	5.46	5.44
S. pombe	2- Oliva et al., 2005 elut1	1.93	1.81	1.82	4.10	4.10	0.84	1.42	1.95	3.33	1.04	1.69
S. pombe	3- Oliva et al., 2005 elut2	4.86	5.32	6.20	1.06	1.02	3.60	5.01	5.64	5.09	4.23	3.85
S. pombe	4- Peng et al., 2005 cdc	1.59	2.23	1.78	4.92	5.09	3.51	3.09	4.25	0.63	1.96	2.95
S. pombe	5- Peng et al., 2005 elut	4.33	4.71	5.32	5.06	5.27	4.17	4.11	5.12	3.35	4.25	5.03
S. pombe	6- Rustici et al., 2004 cdc1	6.04	0.13	0.51	3.18	3.15	6.14	1.78	0.02	0.67	1.35	0.57
S. pombe	7- Rustici et al., 2004 cdc2	6.27	0.04	0.12	3.84	3.70	2.56	3.34	4.94	1.01	1.98	1.68
S. pombe	8- Rustici et al., 2004 elut1	1.56	2.28	2.51	2.82	2.85	1.95	1.80	1.97	1.19	1.83	2.26
S. pombe	9- Rustici et al., 2004 elut2	1.83	1.61	0.86	2.99	2.80	1.48	2.38	1.60	1.01	1.75	3.20
S. pombe	10- Rustici et al., 2004 elut3	3.25	2.91	4.06	3.47	3.47	3.14	3.71	2.52	2.88	2.34	3.55
S. cerevisiae	1- Cho at al. 1998	3.91	3.50	2.57	3.30	3.44	3.03	3.50	3.90	4.30	4.40	1.99
S. cerevisiae	2- De Lichtenberg et al., 2005	4.90	4.00	2.15	1.25	2.09	3.66	4.00	4.49	4.94	5.34	2.93
S. cerevisiae	3- Pramila et al., 2006 30	3.03	1.07	3.03	3.28	3.32	3.98	3.79	3.21	4.83	5.58	2.58
S. cerevisiae	4- Pramila et al., 2006 38	3.65	1.88	2.89	3.51	3.79	4.08	4.28	3.21	4.62	5.52	2.56
S. cerevisiae	5- Spellman et al., 1998 alpha	3.05	2.49	2.73	3.34	3.64	3.74	3.99	3.09	4.46	5.86	2.98
S. cerevisiae	6- Spellman et al., 1998 cdc	5.48	0.84	2.69	3.54	3.87	4.49	4.21	5.41	5.55	6.06	2.51
Humans	1- Whitfield et al., 2002 Thynoc	2.78	1.96	2.50	3.28	4.05	0.36	0.78	6.04	0.69	5.12	0.83
Humans	2- Whitfield et al., 2002 Thy1	4.62	3.41	3.44	3.49	3.54	1.47	1.11	5.35	0.16	5.82	0.43
Humans	3- Whitfield et al., 2002 Thy2	4.43	4.25	4.85	4.94	5.03	1.40	1.18	0.16	0.12	6.13	0.24
Humans	4- Whitfield et al., 2002 Thy3	3.11	3.60	4.47	4.21	5.13	0.34	0.48	0.11	0.41	5.88	1.46

Table B: Values of concentration parameter (κ) according to experiments

Species	Experiment									
	1	2	3	4	5	6	7	8	9	10
S. pombe	1.63	1.54	1.61	1.08	9.08	1.40	0.14	26.86	2.52	3.46
S. cerevisiae	1.80	0.84	10.47	26.64	8.80	1.79				
Human	1.72	2.34	26.78	2.46						

References

[1] Liu D, Umbach DM, Peddada SD, Li L, Crockett PW, Weinberg CR. 2004. A random periods model for expression of cell-cycle genes. *Proc. Natl. Acad. Sci. USA* 101(19):7240-7245.

[2] Fernández MA, Rueda C, Peddada, SD. 2012. Identification of a core set of signature cell-cycle genes whose relative order of time to peak expression is conserved across species. *Nucl. Acids Res.* 40(7):2823-2832.

[3] Oliva A, Rosebrock A, Ferrezuelo F, Pyne S, Chen H, Skiena S, Futcher B, Leatherwood J. 2005. The cell-cycle-regulated genes of Schizosaccharomyces pombe. *PloS Biology* 3:1239-1260.

[4] Peng X, Karutury RKM, Miller LD, Kui L, Yonghui J, et al. 2005. Identification of cell-cycle-regulated genes in fission yeast. *Mol. Biol. Cell* 16:1026-1042.

[5] Rustici G, Mata J, Kivinen K, Lió P, Penkett CJ, Burns G, Hayles J, Brazma A, Nurse P, Bähler J. 2004. Periodic gene expression program of the fission yeast cell-cycle. *Nature Genetics* 36:809-817.

[6] Cho RJ, Campbell MJ, Winzeler EA, Steinmetz L, Conway A, et al. 1998. A genome-wide transcriptional analysis of the mitotic cell-cycle. *Mol. Cell.* 2(1):65-73.

[7] De Lichtenberg U, Wernersson R, Jensen TS, Nielsen HB, Fausbøll A, Schmidt P, Hansen FB, Knudsen S, Brunak S. 2005. New weakly expressed cell cycle-regulated genes in yeast. *Yeast* 22(5):1191-1201.

[8] Pramila T, Wu W, Miles S, Noble WS, Breeden LL. 2006. The forkhead transcription factor Hcm1 regulates chromosome segregation genes and fills the S-phase gap in the transcriptional circuitry of the cell cycle. *Genes Dev.* 22(16):2266-2278.

[9] Spellman PT, Sherlock G, Zhang MQ, Iyer VR, Anders K, et al. 1998. Comprehensive identification of cell cycle-regulated genes of the yeast Saccharomyces cerevisiae by microarray hybridization. *Mol. Biol. Cell.* 9(12):3273-3297.

[10] Whitfield ML, Sherlock G, Saldanha AJ, Murray JI, Ball CA, et al. 2002. Identification of genes periodically expressed in the human cell-cycle and their expression in tumors. *Mol. Biol. Cell.* 13:1977-2000.
