# Supplementary material for: Determination of Temporal Order among the Components of an Oscillatory System
Source: PLoS One. 2015 Jul 7;10(7):e0124842. doi: 10.1371/journal.pone.0124842 (PMC4495067; doi:10.1371/journal.pone.0124842)
Supplement: S3 File — (PDF) [file pone.0124842.s003.pdf]

Operating characteristics of the test statistic

1 Study design

Using a variety of synthetic data we evaluated the false positive rate (Type I error) and power of the proposed test statistic. Our simulation experiment mimicked the cell-cycle experiment data described in this paper. Thus we generated synthetic data representing 20 experiments on 3 artificial species with 10 experiments corresponding to species S_1 (similar to *S. pombe*), 6 corresponding to species S_2 (similar to *S. cerevisiae*) and 4 corresponding species S_3 (similar to human Hela cell line data). We considered 4 patterns (N_1, N_2, N_3 and N_4) of phase angle parameters representing the null hypotheses and 3 patterns (A_1, A_2 and A_3) representing the alternative hypotheses. Let ϕ_i^s denote the phase angle of the ith gene, $i = 1, 2, \ldots G$ in species $s = S_1, S_2, S_3$. In this simulation experiment we considered $G = 6$ cell-cycle genes. Corresponding to each ϕ_i^s, we generated the estimated phase angle $\hat{\phi}_i^s$ according to independent von-Mises distribution with angular mean direction ϕ_i^s and concentration parameter κ_j. Patterns of ϕ_i^s are described below while concentration parameters κ_j appear in Table B in S2 File. Corresponding to each pattern we generated 100 runs to estimate the Type I error and power. The nominal Type I error rate was taken to be 0.05.

1.1 Patterns of null hypothesis

For all patterns of null hypotheses except N_3 described below, we take $\phi_i^{S_1} = \phi_i^{S_2} = \phi_i^{S_3} = \phi_i$, for all $i = 1, 2, \ldots, 6$. Values of concentration parameter κ for patterns N_1, N_2 and N_3 were obtained from the 20 real data sets in Table S2. Values of κ for N_4 are defined below.

Pattern N_1: $\phi =$ (1.57, 2.28, 2.51, 2.82, 2.85, 1.19)'. These values have been taken from unconstrained estimates for *S. pombe* 8- Rustici et al. (2004) elut1 experiment, see Table A in S2 File.

Pattern N_2: $\phi =$ (1.37, 2.60, 2.60, 2.60, 2.60, 1.37)' which are the circular means of the ϕ values in Pattern N_1 divided into two groups. Thus we are introducing equalities in the phase angles.

Pattern N_3: In this case although the components of ϕ satisfy the same order among the 6 genes in the three artificial species, the ϕ are not same for all three species. They are as follows: $\phi^{S_1} =$ (1.05, 2.09, 3.14, 4.19, 5.23, 6.28)', uniformily distributed in the interval $(0, 2\pi)$, $\phi^{S_2} =$ (0.13, 0.26, 0.39, 0.52, 0.65, 0.78)', uniformily distributed in the interval $(0, \pi/4)$ and $\phi^{S_3} =$ (3.21, 3.27, 3.34, 3.40, 3.47, 3.53)',

uniformily distributed in the interval $(\pi, 9\pi/8)$. Thus even though $\phi^{S_1} \neq \phi^{S_2} \neq \phi^{S_3}$, the order of phase angles is the same, i.e. $O_{S_1} = O_{S_2} = O_{S_3}$.

Pattern N_4**:** Although, as in patterns N_1 and N_2, ϕ was same for all three species with $\phi^{S_1} = \phi^{S_2} = \phi^{S_3} = \phi =$ (1.57, 2.28, 2.51, 2.82, 2.85, 1.19)', the values of the concentration parameters κ were chosen so that the four largest concentration parameter were assigned to the 4 experiments in species S_3, the next six largest concentration parameters were assigned to the 6 experiments in S_2 and the smallest ten concentration parameters were assigned to the 10 experiments in S_1. Thus we are introducing heterogeneity among the three species in terms of concentration parameters.

1.2 Patterns of alternative hypothesis

We generated 3 different patterns of ϕ: $\phi^{S_1}, \phi^{S_2}, \phi^{S_3}$ for the three artificial species as described below. As in the case of null hypothesis, patterns of κ for Pattern A_1 and A_2 were obtained from the 20 real data sets in Table S2.

Pattern A_1**:** $\phi^{S_1} =$ (1.57, 2.28, 2.51, 2.82, 2.85, 1.19)', from *S. pombe* 8- Rustici et al. (2004) elut1 experiment, $\phi^{S_2} =$ (4.02, 0.10, 2.15, 1.25, 2.09,4.68)' from *S. cerevisiae* 2- De Lichtenberg et al. (2002) experiment and $\phi^{S_3} =$ (2.78, 1.96, 2.69, 1.33, 4.05, 0.69) from *Humans* 1- Whitfield et al. (2002) Thynoc experiment. Thus in this case the order of expression of the 6 genes in the three species are given by: $O_{S_1} = \{1, 2, 3, 4, 5, 6\}$, $O_{S_2} = \{1, 6, 2, 4, 5, 3\}$, and $O_{S_3} = \{1, 5, 6, 4, 2, 3\}$.

Pattern A_2**:** $\phi^{S_1} =$ (1.57, 2.28, 2.51, 2.82, 2.85, 1.19)', from *S. pombe* 8- Rustici et al. (2004) elut1 experiment, $\phi^{S_2} =$ (0.00, 0.75, 5.77, 0.68, 5.69, 1.12)', from *S. pombe* 1- Oliva et al. (2005) cdc experiment and $\phi^{S_3} =$ (0.04, 6.27, 0.12, 3.84, 3.70, 1.01)', from *S. pombe* 7- Rustici et al. (2004) cdc2 experiment. Thus the order of expression of the 6 genes in the three species are given by: $O_{S_1} = \{1, 2, 3, 4, 5, 6\}$, $O_{S_2} = \{1, 4, 2, 6, 5, 3\}$, and $O_{S_3} = \{1, 3, 6, 5, 4, 2\}$.

Pattern A_3**:** Patterns of ϕ are same as those of Pattern A_1 but as in the null pattern N_4 the values of the concentration parameters κ were chosen so that the four largest concentration parameter were assigned to the 4 experiments in species S_3, the next six largest concentration parameters were assigned to the 6 experiments in S_2 and the smallest ten concentration parameters were assigned to the 10 experiments in S_1. Thus we are introducing heterogeneity among the three species in terms of concentration parameters.

2 Results

As seen from the results of our simulation experiment summarized in Table A, the proposed test never exceeds the false positive rate of 0.05 by more than a standard error and is sufficiently powerful even for mild departures from the null hypothesis.

Table A: Results of a simulation study.

Case	Pattern	Probability of rejection the null hypothesis
Type I error	Null N_1	0.01
	Null N_2	0.02
	Null N_3	0.055
	Null N_4	0.04
Power	Alternative A_1	1
	Alternative A_2	0.93
	Alternative A_3	0.85

References

[1] Rustici G, Mata J, Kivinen K, Lió P, Penkett CJ, Burns G, Hayles J, Brazma A, Nurse P, Bähler J. 2004. Periodic gene expression program of the fission yeast cell-cycle. *Nature Genetics* 36:809-817.

[2] De Lichtenberg U, Wernersson R, Jensen TS, Nielsen HB, Fausbøll A, Schmidt P, Hansen FB, Knudsen S, Brunak S. 2005. New weakly expressed cell cycle-regulated genes in yeast. *Yeast* 22(5):1191-1201.

[3] Whitfield ML, Sherlock G, Saldanha AJ, Murray JI, Ball CA, et al. 2002. Identification of genes periodically expressed in the human cell-cycle and their expression in tumors. *Mol. Biol. Cell.* 13:1977-2000.

[4] Oliva A, Rosebrock A, Ferrezuelo F, Pyne S, Chen H, Skiena S, Futcher B, Leatherwood J. 2005. The cell-cycle-regulated genes of Schizosaccharomyces pombe. *PloS Biology* 3:1239-1260.
