# Supplementary material for: Determination of Temporal Order among the Components of an Oscillatory System
Source: PLoS One. 2015 Jul 7;10(7):e0124842. doi: 10.1371/journal.pone.0124842 (PMC4495067; doi:10.1371/journal.pone.0124842)
Supplement: S4 File — (PDF) [file pone.0124842.s004.pdf]

Gene forward selection procedure and results

The purpose of this file is to detail the selection procedure of a maximum size set of genes whose order is common in different species and the results obtained. Table 1 in the main paper contains the names of the genes and their orthologs/paralogs in the different species as well as the number assigned to each gene to ease the specification of the circular order obtained. We start trying to find the maximum set of genes which have a common circular order among the three species (*S.pombe*, *S.cerevisiae* and Humans). As we mentioned in the main document we used the four human data obtained from Whitfield et al. (2002); six budding yeast data (one from Cho et al. (1998), another from De Lichtenberg et al. (2005), two from Pramila et al. (2006) and two from Spellman et al. (1998) and ten fission yeast data (five by Rustici et al. (2004), three by Oliva et al. (2005) and two by Peng et al. (2005).

As three is the minimum number of elements with possibly different circular orders, we have checked all possible triples of the 11 available genes (165 triples) and the two possible circular orders in each triple, (i.e. 165·2 circular orders) calculating the MSCE between each order and the whole dataset (20 experiments). We found that there were five subsets of three genes for which the same minimum value of the MSCE was attained: (2, 4, 5); (4, 5, 6); (4, 5, 7); (4, 5, 10); (4, 5, 11) with MSCE=0.000057. Then, to split this draw, we added a fourth gene to each of these five subsets. When we did this a single set (2, 4, 5, 6) was obtained, with minimum MSCE=0.000991 for the order $cdc18 \preceq hhf1 \preceq hta2 \preceq fhk2 \preceq cdc18$. When tested, this order had a p-value of 0.153 and a confidence coefficient of 100%, i.e. this order was always obtained when the resampling procedure was run.

We use this set to start the forward procedure for the three species. In each step, the best gene to be added is searched. One gene is added at a time and the test of equality of circular orders is executed with the following criterion.

We discard genes whose inclusion yields a p-value≤0.1 in the test of order comparison. For those genes which result in a p-value>0.1, we obtain the Confidence Coefficient (CC) for the candidate order, i.e. a circular order (simple or partial) coherent with the MFO (most frequent order) as well as coherent with the candidates in previous steps. Among the genes with CC>70%, the one with highest confidence coefficient is chosen.

We repeated the above procedure by adding a gene at a time until we discovered the best subset of genes meeting the established criteria. Once there are no more genes to be added, we have obtained the

maximum set of common genes among the three species. These results appear in Table A below.

Table A: 3 species comparison

N. Genes	P-value	Simple order	CC	Partial order	CC
4	0.153	$2\preceq 4\preceq 5\preceq 6\preceq 2$	100		
5	0.414	$2\preceq 3\preceq 4\preceq 5\preceq 6\preceq 2$	99.85		
6	0.488	$2\preceq 3\preceq 4\preceq 5\preceq 7\preceq 6\preceq 2$	76.06	$2\preceq 3\preceq 4\preceq 5\preceq [6,7]\preceq 2$	100

Now we analyze the species by pairs considering the criterion we have established and using as starting set the the set of genes obtained in the comparison of the three species. The results of this forward procedure for each pair of species *S.pombe*-*S.cerevisiae*, *S.pombe*-Humans and *S.cerevisiae*-Humans appear in Tables B, C and D respectively.

Table B: *S.pombe*-*S.cerevisiae* comparison

N. Genes	P-value	Simple order	CC	Partial order	CC
6	0.34	$2\preceq 3\preceq 4\preceq 5\preceq 7\preceq 6\preceq 2$	60.65	$2\preceq 3\preceq 4\preceq 5\preceq [6,7]\preceq 2$	99.15
7	0.188	$2\preceq 3\preceq 4\preceq 5\preceq 6\preceq 7\preceq 10\preceq 2$	63.76	$2\preceq 3\preceq 4\preceq 5\preceq [6,7]\preceq 10\preceq 2$	99.65
8	0.619	$2\preceq 11\preceq 3\preceq 4\preceq 5\preceq 6\preceq 7\preceq 10\preceq 2$	77.51	$2\preceq 11\preceq 3\preceq 4\preceq 5\preceq [6,7]\preceq 10\preceq 2$	85.61
9	0.866	$2\preceq 11\preceq 3\preceq 4\preceq 5\preceq 1\preceq 6\preceq 7\preceq 10\preceq 2$	67.1	$2\preceq 11\preceq 3\preceq [1,4,5]\preceq [6,7]\preceq 10\preceq 2$	73.96
10	0.336	$2\preceq 11\preceq 3\preceq 4\preceq 5\preceq 8\preceq 1\preceq 6\preceq 7\preceq 10\preceq 2$	25.98	$2\preceq 11\preceq 3\preceq [1,4,5,8]\preceq [6,7]\preceq 10\preceq 2$	72.31

Table C: *S.pombe*-Humans comparison

N. Genes	P-value	Simple order	CC	Partial order	CC
6	0.664	$2\preceq 3\preceq 4\preceq 5\preceq 7\preceq 6\preceq 2$	87.5	$2\preceq 3\preceq 4\preceq 5\preceq [6,7]\preceq 2$	99.5
7	0.72	$2\preceq 3\preceq 4\preceq 5\preceq 9\preceq 6\preceq 7\preceq 2$	82.42	$2\preceq 3\preceq 4\preceq 5\preceq 9\preceq [6,7]\preceq 2$	97.27
8	0.437	$1\preceq 2\preceq 3\preceq 4\preceq 5\preceq 9\preceq 6\preceq 7\preceq 2$	45.75	$[1,2]\preceq 3\preceq 4\preceq 5\preceq 9\preceq [6,7]\preceq 2$	92.6

Table D: *S.cerevisiae*-Humans comparison

N. Genes	P-value	Simple order	CC	Partial order	CC
6	0.119	$2\preceq 3\preceq 4\preceq 5\preceq 6\preceq 7\preceq 2$	42.25	$2\preceq 3\preceq 4\preceq 5\preceq [6,7]\preceq 2$	99.15

References

[1] Whitfield ML, Sherlock G, Saldanha AJ, Murray JI, Ball CA, et al. 2002. Identification of genes periodically expressed in the human cell-cycle and their expression in tumors. *Mol. Biol. Cell.* 13:1977-2000.

[2] Cho RJ, Campbell MJ, Winzeler EA, Steinmetz L, Conway A, et al. 1998. A genome-wide transcriptional analysis of the mitotic cell-cycle. *Mol. Cell.* 2(1):65-73.

[3] De Lichtenberg U, Wernersson R, Jensen TS, Nielsen HB, Fausbøll A, Schmidt P, Hansen FB, Knudsen S, Brunak S. 2005. New weakly expressed cell cycle-regulated genes in yeast. *Yeast* 22(5):1191-1201.

[4] Pramila T, Wu W, Miles S, Noble WS, Breeden LL. 2006. The forkhead transcription factor Hcm1 regulates chromosome segregation genes and fills the S-phase gap in the transcriptional circuitry of the cell cycle. *Genes Dev.* 22(16):2266-2278.

[5] Spellman PT, Sherlock G, Zhang MQ, Iyer VR, Anders K, et al. 1998. Comprehensive identification of cell cycle-regulated genes of the yeast Saccharomyces cerevisiae by microarray hybridization. *Mol. Biol. Cell.* 9(12):3273-3297.

[6] Rustici G, Mata J, Kivinen K, Lió P, Penkett CJ, Burns G, Hayles J, Brazma A, Nurse P, Bähler J. 2004. Periodic gene expression program of the fission yeast cell-cycle. *Nature Genetics* 36:809-817.

[7] Oliva A, Rosebrock A, Ferrezuelo F, Pyne S, Chen H, Skiena S, Futcher B, Leatherwood J. 2005. The cell-cycle-regulated genes of Schizosaccharomyces pombe. *PloS Biology* 3:1239-1260.

[8] Peng X, Karutury RKM, Miller LD, Kui L, Yonghui J, et al. 2005. Identification of cell-cycle-regulated genes in fission yeast. *Mol. Biol. Cell* 16:1026-1042.
